# Atypical Guillain-Barré Syndrome in a Young Adult: Diagnostic Challenges and Lessons Learned

**DOI:** 10.7759/cureus.82731

**Published:** 2025-04-21

**Authors:** Hannah Becker, Edena R Khoshaba, David A Ansell

**Affiliations:** 1 Medical School, Rush University Medical Center, Chicago, USA; 2 Internal Medicine, Rush University Medical Center, Chicago, USA

**Keywords:** atypical presentation, electromyography, guillain-barré syndrome, intravenous immunoglobulin, primary care, sensory predominance

## Abstract

Guillain-Barré Syndrome (GBS) is a rare, immune-mediated polyneuropathy that typically presents with ascending weakness, areflexia, and sensory disturbances. This case highlights an atypical presentation of GBS in an 18-year-old female, whose initial symptoms included primarily sensory deficits following routine exercise. The diagnostic process was complicated by unremarkable initial laboratory and imaging findings, but cerebrospinal fluid analysis revealed albuminocytologic dissociation, and electromyography confirmed acute demyelinating polyneuropathy. This case underscores the diagnostic challenges posed by atypical variants of GBS, the importance of thorough neurological evaluation, and the critical role of timely intervention in optimizing outcomes.

## Introduction

Guillain-Barré syndrome (GBS) is a rare, immune-mediated polyneuropathy that typically presents with rapidly progressive, symmetrical ascending weakness, areflexia, and sensory disturbances [1]. It is the most common cause of acute neuromuscular paralysis worldwide, with around 100,000 new cases each year [2]. The pathophysiology is thought to be an immune-mediated disorder in which the body’s immune system produces antibodies and inflammatory cells that mistakenly target the peripheral nerves and nerve roots through molecular mimicry [3]. This immune attack, often prompted by a preceding infection, results in either demyelination, axonal damage, or both, disrupting normal nerve function [3].

While the classic presentation of GBS is well-recognized, atypical manifestations can pose significant diagnostic challenges, particularly in young, otherwise healthy individuals. For instance, Miller Fisher syndrome is a rare variant of GBS characterized by the clinical triad of ophthalmoplegia, ataxia, and areflexia [4]. Pure sensory GBS exhibits an acute onset of sensory neuropathy without prominent muscle weakness [5]. Clinical features include the sudden onset of sensory loss, diminished or absent reflexes, and electrophysiological evidence of demyelination in sensory nerves. Other variants include disease that mimics brainstem stroke with cranial nerve palsies and autonomic dysfunction [6].

Early recognition of GBS is critical, as timely initiation of therapies such as intravenous immunoglobulin (IVIG) or plasma exchange can significantly improve outcomes and prevent severe complications, including respiratory failure and dysautonomia.

## Case presentation

We present the case of an 18-year-old female patient with no significant past medical history who came to an outpatient clinic with a one-week history of neurological symptoms. She reported perioral and tongue tingling, bilateral leg numbness, tingling, weakness, and difficulty climbing stairs. The symptoms began after a routine run, which was not more strenuous than her typical runs. Initially, she experienced calf soreness, which she attributed to exercise. However, later that day, she developed numbness in her feet that gradually ascended to her calves and thighs. By evening, she noticed a slower walking pace and progressive worsening of her symptoms.

Her first physical exam in the office showed that she was alert, oriented, and demonstrated normal memory, attention, language, and overall cognitive function. The cranial nerve examination was unremarkable, with intact visual fields and acuity, equal and reactive pupils, normal extraocular movements without diplopia, and preserved facial sensation, movement, hearing, and palate elevation, as well as intact shoulder shrug and tongue function. Motor strength was full (5/5) in the neck and all extremities, though she required the use of her arms to rise from a chair, suggesting some functional limitation. Sensory testing revealed patchy, non-dermatomal loss predominantly affecting the feet and big toe with tuning fork and monofilament assessment, despite normal light touch sensation in the arms and legs. Reflexes were symmetric bilaterally, and the Babinski sign was absent. Coordination tests were unremarkable; however, her gait was unsteady compared to that of a young adult, with marked difficulty performing tandem gait and Romberg, resulting in falls and a sensation of imbalance. The patient was counseled on warning signs that would require evaluation in the emergency department; since the patient had just been to an outside hospital, she opted to monitor her symptoms at home. 

Over the following days, her symptoms persisted, with increasing numbness, making it difficult for her to walk normally or ascend stairs. She attempted to run again but was only able to manage a few blocks. The numbness extended to her fingers, mouth, and tongue, causing significant discomfort and prompting her to seek medical attention.

The patient initially presented to the emergency department at another hospital. A neurological examination and laboratory workup were unremarkable, including tests for Lyme disease, HIV, hepatitis C, RPR, vitamin B12, CBC, and CMP. Imaging was not performed, and she was discharged without a definitive diagnosis.

The patient also reported an unspecified, mild upper respiratory infection with a cough and sore throat preceding the onset of her neurological symptoms, although she could not recall the exact timing. She denied back pain, visual disturbances, headaches, nausea, vomiting, fatigue, or unexplained weight loss. She had spent part of the summer camping in the Northeast region. She had no recent immunizations. 

A few days later, she experienced an episode of urinary incontinence, prompting hospital admission. On examination, she was found to have absent bilateral lower extremity reflexes and decreased sensation to pinprick, temperature, vibration, and proprioception in both legs. Records from the outside hospital were reviewed, and additional testing was ordered, including a respiratory pathogen panel, which was negative. Autoimmune profile (ANA screen, rheumatoid factor, CRP, C3/C4), ganglioside GM-1 IgG and IgM antibodies, and anti-neutrophil cytoplasmic antibodies were all unremarkable. 

**Figure 1:**
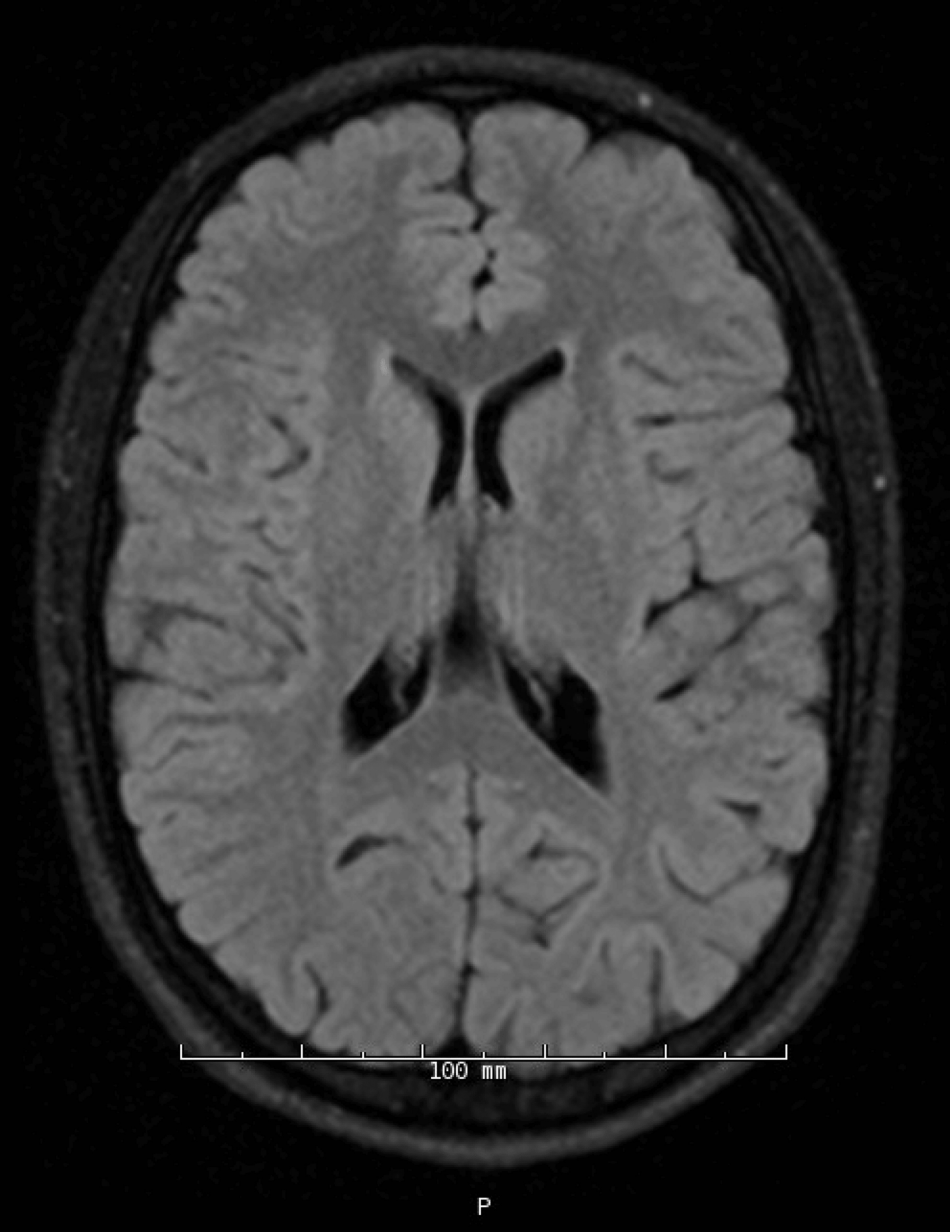
T2/FLAIR MRI brain without IV contrast

**Figure 2:**
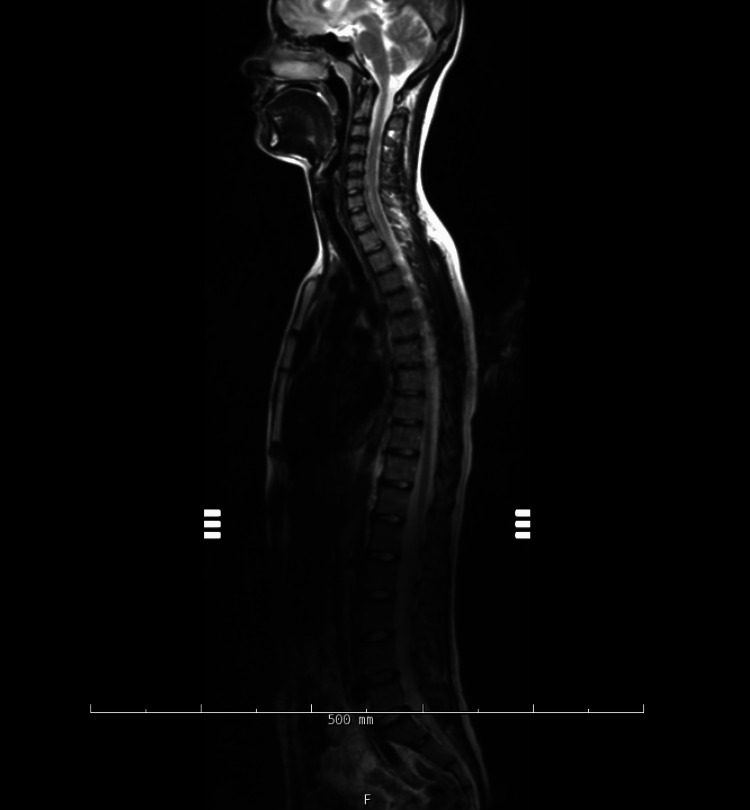
T2 MRI entire spinal cord without IV contrast

During her hospital course, an MRI of the brain, and cervical/thoracic/lumbar spine without contrast showed no abnormalities as seen in Figures 1-2.

Lumbar puncture revealed a cerebrospinal fluid (CSF) protein level of 103.7 mg/dL (ref 15.0-45.0 mg/dL) and glucose of 89 mg/dL (ref 45-70 mg/dL), raising suspicion for GBS despite the atypical presentation. Additionally, the EBV capsid antigen IgM in the CSF was detected. These values for the CSF studies can be seen in Table 1. 

**Table 1:** CSF studies supporting Guillain-Barré syndrome diagnosis CSF: cerebrospinal fluid; RBC: red blood cells; WBC: white blood cells; H: high

CSF Cell Count/Differential	Findings
Color	Colorless
Clarity	Clear
Xanthochromia	None seen
Gross blood	None seen
WBC	3
RBC	1
Lymphocyte	1
Glucose	89 (H)
Protein	103.7 (H)
Oligoclonal bands	None seen
EBV capsid antigen IgM	1.0 (H)

A neuromuscular specialist was consulted, and electromyography confirmed findings consistent with acute demyelinating polyneuropathy (Table 2). 

**Table 2:** Summary of electromyogram and nerve conduction studies APB: abductor pollicis brevis; ADM: abductor digiti minimi, EDB: extensor digitorum brevis; AH: abductor hallicus

Nerve	Side	Test Type	Findings	Interpretation
Median	Left	Motor (APB)	↑ Latency (7.97ms, ref ≤4.40ms), ↓ Amplitude (1.7mV, ref ≥4.0mV)	Prolonged latency and decreased amplitude of the left median motor response
Median	Left	Sensory (Digit II)	Normal parameters, Onset: 1.93ms, Peak: 2.71ms, Amplitude: 21.9μV	Preserved sensory function
Ulnar	Left	Motor (ADM)	↑ Latency (4.06ms, ref ≤3.60ms), ↓ Amplitude (4.4mV, ref ≥5.0mV), Slowed conduction across elbow	Prolonged latency and decreased amplitude of the left ulnar motor response
Ulnar	Left	Sensory (Digit V)	Absent response	Complete sensory block
Peroneal	Left	Motor (EDB)	↑↑ Latency (12.08ms, ref ≤6.20ms), ↓↓ Amplitude (1.1mV, ref ≥2.0mV), ↓ Conduction velocity (35m/s, ref ≥39m/s)	Prolonged latency and decreased amplitude of the left peroneal motor response
Tibial	Left	Motor (AH)	↑↑ Latency (11.09ms, ref ≤6.00ms), ↓ Amplitude (1.6mV, ref ≥3.0mV), ↑ F-wave latency	Prolonged latency and decreased amplitude of the left tibial motor response
Sural	Left	Sensory	Normal parameters	Preserved
Superficial Peroneal	Left	Sensory	Normal parameters	Preserved
All tested nerves	Right	Motor and Sensory	Normal parameters	No abnormalities

The above studies revealed predominantly motor abnormalities characterized by prolonged distal latencies and decreased amplitudes in multiple left-sided nerves. Specifically, the findings indicated significant abnormalities in the left median, ulnar, peroneal, and tibial motor responses, with the peroneal and tibial nerves showing the most severe changes. 

The patient was started on intravenous immunoglobulin (IVIG) at a dose of 2 g/kg, divided over four days (30 g/day). After the completion of her therapy, she was discharged with a plan for outpatient follow-up with her primary care provider, neuromuscular clinic, and physical therapy. At the time of discharge, she was still endorsing minimal persistent paresthesias throughout her legs. 

## Discussion

This case highlights an atypical presentation of GBS in a young, otherwise healthy individual, underscoring the challenges that arise when the clinical course deviates from the classic description. While the hallmark features of GBS (symmetrical ascending weakness, areflexia, and sensory disturbances) were ultimately present, the patient’s initial presentation, characterized by perioral tingling and calf soreness following routine exercise, obscured the diagnosis. The delayed recognition of GBS in this case reflects the complexity of identifying rare variants or early manifestations of the disease.

The patient’s gradual progression of symptoms, including ascending numbness, weakness, and eventual urinary incontinence, mirrors the typical disease trajectory of GBS but with unusual features such as prominent sensory symptoms in the absence of overt weakness during the initial stages. This pattern is consistent with sensory-predominant variants of GBS, which are less frequently encountered and can lead to diagnostic uncertainty [6]. The absence of imaging abnormalities and the unremarkable initial laboratory workup further complicated the diagnostic process, emphasizing the importance of a thorough neurological assessment and clinical suspicion in guiding the workup.

A key diagnostic milestone in this case was the CSF analysis, which revealed albuminocytologic dissociation (elevated protein with normal white blood cell count), a classic but nonspecific finding in GBS [7]. Electromyographic studies confirmed acute demyelinating polyneuropathy due to the presence of prolonged latency and decreased amplitude in the motor response of many nerves in her left upper and lower extremities. Interestingly, while the patient presented with sensory symptoms, the electrophysiologic findings show primarily motor involvement, creating a clinical-electrophysiological dissociation. This dissociation is atypical, as patients with GBS usually present with symptoms matching their neurophysiological findings. Only the left ulnar sensory response is absent, while other sensory nerves (median, radial, sural, and superficial peroneal) show preserved function, despite the patient's sensory complaints. This pattern of motor-predominant involvement with minimal sensory abnormalities on testing is characteristic of the acute inflammatory demyelinating polyneuropathy variant of GBS, though the presentation with sensory symptoms creates a diagnostic curiosity. These findings reiterate the pivotal role of ancillary testing, particularly when the clinical picture is ambiguous. The delayed presentation to the hospital highlights a common challenge in GBS, where the insidious onset of symptoms can lead to delays in seeking medical attention.

This case also underscores the need to consider GBS even in patients with subtle or atypical neurological symptoms, particularly when there is a history of preceding illness, as in this patient’s case with a recent upper respiratory infection. *Campylobacter jejuni* is the most commonly identified trigger of GBS, though a variety of other infections, including viral illnesses, have been implicated, such as cytomegalovirus (CMV), Epstein-Barr virus (EBV), herpes simplex virus (HSV), Lyme disease, and Zika virus [1]. EBV was identified through positive CSF testing for this patient, which was ultimately deemed the likely precipitating factor in this patient’s illness.

IVIG administered promptly after diagnosis remains a cornerstone of therapy for acute demyelinating polyneuropathies, with demonstrated efficacy in halting disease progression and promoting recovery [4]. The patient's improvement following a standard IVIG course highlights the importance of early intervention, even in atypical cases where diagnostic uncertainty might initially delay treatment.

Moreover, this case emphasizes the necessity of a thorough neurological examination, including assessment of reflexes, strength, sensation, and coordination. The absence of lower extremity reflexes and reduced sensation to pain, temperature, vibration, and proprioception in this patient provided important clinical clues that warranted further investigation despite initial negative findings.

## Conclusions

This case highlights several key lessons for clinicians. GBS is a highly variable condition, and its presentation can be deceptively subtle or atypical. A clinician's ability to maintain a high index of suspicion is crucial, especially in young, healthy individuals presenting with unexplained progressive neurological symptoms. The delay in diagnosis here underscores how easy it is to overlook nonspecific signs, leading to a potential misstep in management. In addition to clinical evaluation, ancillary tests such as cerebrospinal fluid analysis and electromyographic studies play an essential role in confirming GBS and shaping treatment decisions. Ultimately, recognizing this condition early and beginning treatment promptly are the cornerstones of preventing lasting complications and improving patient outcomes. This case reinforces the importance of staying alert to atypical neurological presentations and adopting a thorough, systematic approach to diagnosis and care. GBS may be rare, but its diverse manifestations require clinicians to keep a broad differential and act swiftly to ensure the best possible outcomes.
